# Serum Atrial Natriuretic Peptide, *NPPA* Promoter Methylation, and Cardiovascular Disease: A 10-year Follow-Up Study in Chinese Adults

**DOI:** 10.5334/gh.1116

**Published:** 2022-04-07

**Authors:** Linan Chen, Jing Li, Min Zhang, Qiu Zhang, Lei Wu, Ying Lu, Yan He, Jun Jiang, Xiaolong Zhang, Jianwei Hu, Yi Ding, Mingzhi Zhang, Hao Peng

**Affiliations:** 1 Department of Epidemiology, School of Public Health, Medical College of Soochow University, Suzhou, China; 2 Department of Central Office, Suzhou National New and Hi-tech Industrial Development Zone Center for Disease Control and Prevention, Suzhou, China; 3 Department of Chronic Disease, Gusu Center for Disease Control and Prevention, Suzhou, China; 4 Department of Maternal and Child Health, Suzhou Industrial Park Center for Disease Control and Prevention, Suzhou, China; 5 Jiangsu Key Laboratory of Preventive and Translational Medicine for Geriatric Diseases, Soochow University, Suzhou, China; 6 Department of Tuberculosis Control, Suzhou Center for Disease Control and Prevention, Suzhou, China; 7 Department of Central Office, Maternal and Child Health Bureau of Kunshan, Suzhou, China; 8 Department of Preventive Medicine, College of Clinical Medicine, Suzhou Vocational Health College, Suzhou, China

**Keywords:** atrial natriuretic peptide, cardiovascular disease, prospective cohort study, DNA methylation

## Abstract

**Background:**

Atrial natriuretic peptide (ANP) has been associated with cardiovascular disease (CVD) and related risk factors, but the clinical application is limited and the underlying mechanisms are not very clear. Here, we aimed to examine whether proANP and its coding gene methylation were associated with CVD in the Chinese population.

**Methods:**

Serum proANP and peripheral blood DNA methylation of natriuretic peptide A gene (*NPPA*) promoter was quantified at baseline for 2,498 community members (mean aged 53 years, 38% men) in the Gusu cohort. CVD events were obtained during 10 years of follow-up. A competing-risks survival regression model was applied to examine the prospective associations of proANP and *NPPA* promoter methylation with incident CVD.

**Results:**

During follow-up, 210 participants developed CVD events, 50 participants died from non-cardiovascular causes, and 214 participants were lost. Per 1-nmol/L increment of serum proANP was associated with a 22% (HR = 1.22, 95%CI: 1.03–1.44, *P* = 0.025) higher risk of CVD during follow-up. Of the 9 CpG sites assayed, per 2-fold increment of DNA methylation at CpG3 (located at Chr1:11908299) was significantly associated with a half lower risk of CVD (HR = 0.50, 95%CI: 0.30–0.82, *P* = 0.006). The gene-based analysis found that DNA methylation of the 9 CpGs at *NPPA* promoter as a whole was significantly associated with incident CVD (*P* < 0.05).

**Conclusions:**

Increased proANP and hypomethylation at *NPPA* promoter at baseline predicted an increased future risk of CVD in Chinese adults. Aberrant DNA methylation of the *NPPA* gene may participate in the mechanisms of CVD.

## Introduction

Atrial natriuretic peptide (ANP), a 28-amino-acid peptide hormone produced by the cardiac atria, has been involved in the pathogenesis of cardiovascular disease (CVD) [1,2] and its risk factors e.g., hypertension [3,4], atherosclerosis [5], and diabetes [19,20], through enhancing natriuresis, diuresis, and vasodilatation [6,7]. In addition to these basic findings, the causal role of ANP in CVD incidence has also been suggested by many prospective observational studies [8,9] and clinical trials [10,11]. However, the prospective associations between ANP and the risk of CVD in the Chinese Han population have not been reported. Thus, more studies are needed to elucidate the association between ANP and CVD incidence in Chinese adults. Further, the clinical applications of ANP are limited, although the important role of ANP in CVD has been demonstrated. Synthetic human ANP, carperitide, which has been used in clinical practice for the management of acute decompensated heart failure in Japan [12,13], has some adverse effects such as severe hypotension [13,14,15] and in-hospital death [16,17,18]. It is expected that a better understanding of the molecular mechanisms of the cardiovascular effect of ANP will help its drug development and improvement. Many studies have found that the variants of the coding gene of ANP – *natriuretic peptide A gene* (*NPPA*) were associated with plasma ANP concentrations and CVD [19,20]. For example, polymorphisms in the *NPPA* gene including rs5063, rs5065, and rs5067 have been associated with high risks of stroke [21,22], and coronary artery disease [23,24]. As an interface between the fixed genome and dynamic environment, epigenetic modifications such as DNA methylation play a crucial role in the regulation of transcriptional activity and gene expression [25], and thereby participating in the development of CVD. Indeed, some epigenome-wide association studies have found some methylation markers for CVD [26,27,28]. However, no candidate gene study, to the best of our knowledge, was conducted to examine the association between *NPPA* methylation and CVD. Therefore, leveraging a prospective longitudinal study of Chinese adults in the Gusu cohort, we aimed to examine the associations of ANP and *NPPA* promoter methylation at baseline with incident CVD. The prospective analysis may help causal inference for the role of ANP and *NPPA* promoter methylation in the development of CVD.

## Methods

### Study participants

**Figure 1:**
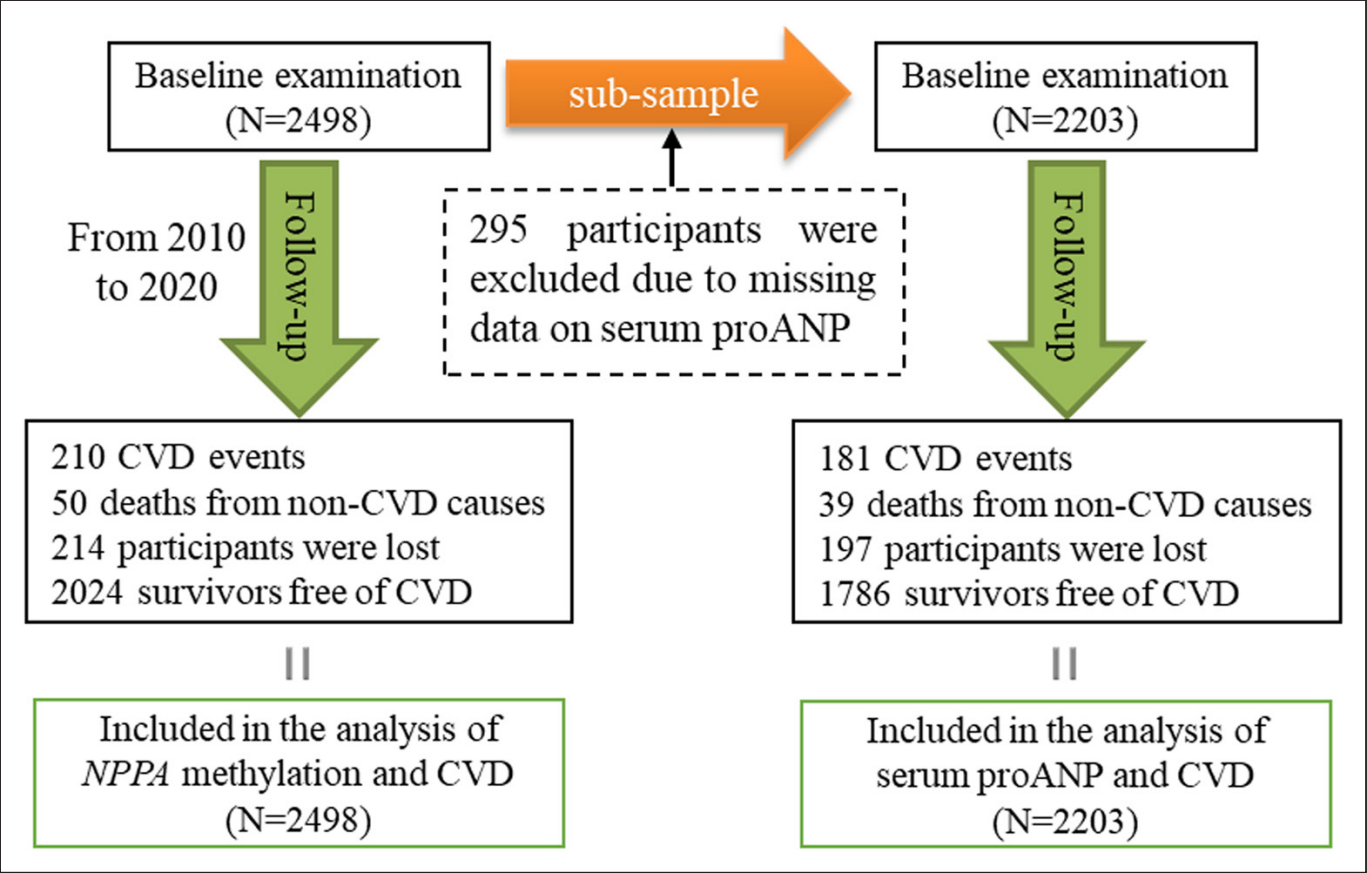
A flowchart illustrating the selection of study participants and statistical plan in the current study. A total of 2498 participants completed the baseline examination in 2010 were followed up for 10 years. During follow-up, 210 participants developed CVD events, 50 participants died from non-cardiovascular causes, and 214 participants were lost. All participants had available data on *NPPA* promoter methylation at baseline and were included in the analysis of the association between NPPA promoter methylation and CVD. Of them, 295 participants were not included in the analysis of the association between proANP and CVD due to missing data on serum proANP at baseline.

The Gusu cohort is a community-based prospective longitudinal study of CVD and its risk factors in middle-aged and elderly Chinese adults. The study design, survey methods, and laboratory techniques have been described previously [29]. In brief, eight communities were randomly selected as the research fields from the 39 communities in Gusu district in 2010. All eligible participants residing in these fields were invited to participate if they were aged over 30 years, with a Han ethnicity, and had lived in the area for at least 10 years. There were a total of 3,061 eligible residents in the study fields, but only 2,706 (participating rate: 88%) individuals agreed to participate in this study. After providing written informed consent, they received questionnaires and were offered free physical examination and clinical biochemical tests using blood and urine specimens under the principle of voluntary acceptance. Based on the information obtained, 208 participants were excluded from the cohort if they met at least one of the following criteria: (1) having clinical suspicion of diseases which may cause secondary hypertension (e.g., renal artery stenosis, coarctation, glomerulonephritis, pyelonephritis, pheochromocytoma, Cushing’s syndrome, Conn’s syndrome), (2) self-reported history of CHD, stroke, or tumors, (3) self-reported thyroid or parathyroid diseases, (4) being pregnant, and (5) lacking blood samples. A total of 2,498 participants completed the baseline examination and were finally enrolled in the Gusu cohort study (***Figure 1***). Hereafter, all participants were followed up every two years for new CVD events through 2020. The protocols were approved by the Soochow University Ethics Committee. Written informed consent was obtained from all study participants.

### Measurement of serum proANP

Blood samples were obtained via venipuncture after an overnight fast for all participants and then stored at –80°C until laboratory tests. Despite being equimolarly cleaved from its precursor protein (1–126 amino acids), the biologically inert proANP (1–98 amino acids; i.e., N-terminal proANP) has a longer half-life and is more stable in the circulation than ANP (99–126 amino acids) [30]. Therefore, we used proANP concentrations to approximately reflect ANP excretion in this study. Serum proANP concentrations were measured using ELISA assays (BIOMEDICA, AUSTRIA), according to the manufacturer’s guidelines. All the samples were processed in a duplicate assay at the Jiangsu Key Laboratory of Preventive and Translational Medicine for Geriatric Diseases by laboratory technicians who were blind to the baseline characteristics of the study participants. Intra- and inter-assay coefficients of variation were less than 2% and less than 4%, respectively.

### Quantification of *NPPA* promoter methylation

**Supplementary Figure S1:**
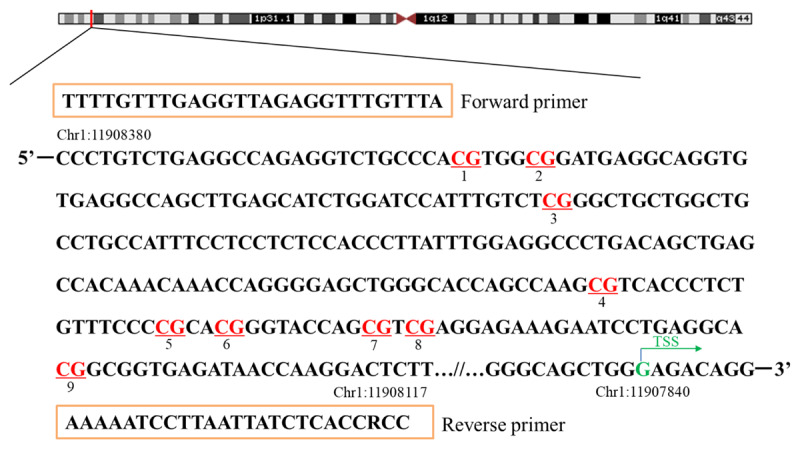
A schematic illustration of the targeted sequence and primers for targeted bisulfite sequencing. Red represents the CpG loci assayed in the NPPA gene promoter (–540 ~ –277 bp from TSS). TSS: transcriptional start site.

DNA methylation levels in the promoter region of the *NPPA* gene were quantified by target bisulfite sequencing as previously described [31], using genomic DNA isolated from peripheral blood mononuclear cells. In brief, based on the genomic coordinates of the *NPPA* promoter in Genome Reference Consortium Human Build 37 (GRCh37), we carefully designed the primers to detect the maximum CpG loci within the CpG islands. The targeted sequence (Chr1: 11908117 – 11908380, reverse strand, relative to TSS: –540 bp to –277 bp) was illustrated in Supplementary ***Figure S1*** and presented previously [31]. Following primer validation, genomic DNA was bisulfite-treated using the EZ DNA Methylation-Gold Kit (Zymo Research, Inc., CA, United States) according to the manufacturer’s protocol, which converts unmethylated cytosine into uracil and leaves methylated cytosine unchanged. The treated samples were amplified, barcoded, and sequenced by Illumina Hiseq 2000 (Illumina, Inc., CA, United States) using the paired-end sequencing protocol according to the manufacturer’s guidelines. The methylation level at each CpG dinucleotide was calculated as the percentage of the methylated alleles over the sum of methylated and unmethylated alleles. For quality control, the samples with bisulfite conversion rate <98% and the cytosine sites with average coverage less than 20× were filtered out. DNA methylation levels were finally quantified at 9 CpG loci in the *NPPA* promoter.

### Follow-up and assessment of cardiovascular events

CVD events in our study included nonfatal coronary heart disease (CHD) including acute myocardial infarction and unstable angina, nonfatal stroke, and death from any CVD causes. All of the participants were followed up by either phone calls or face-to-face visits by staff from the local community health service institutes every two years. The staff reviewed the hospital records and completed a standard event form when a new CHD, stroke, or death was identified during follow-up. Based on the event form, an endpoint review committee made the final diagnosis. The date of each event was ascertained from either the initial point of diagnosis or a death certificate.

### Assessment of risk factors of CVD

Demographic data, including age, sex, and education level, were obtained by questionnaires administered by trained staff. Cigarette smoking was defined as current smoking or not. Current smoking was defined as having smoked at least 100 cigarettes in the entire life, having smoked cigarettes regularly, and smoking currently. Alcohol consumption was classified as current drinkers or not. Current drinkers were those who had consumed any alcoholic beverage ≥12 times during the past year. Three blood pressure measurements were performed by trained staff using a standard mercury sphygmomanometer and a cuff of appropriate size, according to a common protocol adapted from procedures recommended by the American Heart Association [32], after the participants had been resting for at least five min in a relaxed, sitting position. The first and fifth Korotkoff sounds were recorded as systolic blood pressure (SBP) and diastolic blood pressure (DBP), respectively. The means of the three measurements were used for statistical analyses. Body weight (kg) and height (cm) were measured when participants wore light clothes and no shoes by trained staff. Body mass index (BMI) was calculated by dividing weight in kilograms by the square of height in meters (kg/m^2^). Fasting glucose, blood lipids including total cholesterol, triglycerides, high-density lipoprotein cholesterol (HDL-C), and low-density lipoprotein cholesterol (LDL-C), were measured by standard laboratory methods.

### Statistical analysis

To systemically elucidate the role of ANP in the development of CVD, we simultaneously examined the associations of serum proANP and *NPPA* promoter methylation with incident CVD in this study. All statistical analyses were conducted using R version 4.0.2.

### Analysis of the association between serum proANP and CVD

To examine the association between serum proANP and CVD events, we constructed a competing-risks survival regression model in which time (in days) to incident CVD was the dependent variable, serum proANP (continuous proANP or categorical as higher [> the 4^th^ quartile] vs. lower [≤ the 4^th^ quartile] levels) at baseline was the independent variable, and death from causes other than CVD was the competing event, adjusting for age, sex, education level, cigarette smoking, alcohol consumption, BMI, fasting glucose, LDL-C, HDL-C, SBP, and antihypertensive medications at baseline. For participants who were lost or remained free of CVD by the end of follow-up, the time to events was censored at the end of follow-up. The rationale of using the competing-risks survival regression model was to account for the influence of death on the occurrence of CVD events [33], because participants may die for other causes before suffering any CVD events. To ease data interpretation, a partial effect plot with spline curves was captured to visualize the impact of serum proANP level on CVD events by constructing a restricted cubic spline regression model. The competing-risks survival regression models were constructed by the R package cmprsk.’

### Analysis of the association between *NPPA* promoter methylation and CVD

To examine the association between *NPPA* promoter methylation and CVD events, we performed single CpG and gene-based association analyses simultaneously. For single CpG association analysis, we constructed a similar competing-risks survival regression model with DNA methylation level at each CpG locus at baseline (after log2-transformation) as the independent variable. Multiple testing was controlled by adjusting for the total number of CpG loci tested using the false discovery rate (FDR) approach, and an FDR-adjusted *P*-value (i.e., q value) of less than 0.1 was considered statistically significant. For gene-based association analysis, we applied the weighted truncated product method (wTPM) as described previously [34] to examine whether DNA methylation of multiple CpG sites was jointly associated with CVD, based on the results of single CpG association analysis. This method combines *P*-values of all CpGs that reach a preselected threshold (e.g., raw *P* < 0.1 in this study). The regression coefficient of each individual CpG methylation was included as weights in the wTPM statistic. This method has been evaluated by simulation studies [35] and applied to epigenetic analysis [36]. Genotype-Tissue Expression (GTEx) database was applied to examine whether the *NPPA* gene was expressed in white blood cells and target organs of CVD, for example, artery and heart. Integrative DNA methylation (iMethyl) database was applied to examine whether the CpG sites assayed presented in white blood cells and regulated gene expression [37].

## Results

### Baseline characteristics of study participants

The current study included 2,498 (mean aged 53 years, 39% males) middle-aged and elderly Chinese adults. Of them, 295 participants were not included in the analysis of the association between proANP and CVD due to missing data on serum proANP at baseline. Their clinical characteristics at baseline are shown in ***Table 1***. The average level of serum proANP was 1.20 nmol/L, ranging from 0.01 to 5.79 nmol/L. Despite education level and fasting glucose, there was no significant difference in other variables between participants with and without proANP (all *P* < 0.05). The probable selection bias may be unlikely to affect our results.

**Table 1:** Baseline characteristics of study participants in the Gusu cohort study.


CHARACTERISTICS	MEAN ± SD/n (%)			

	TOTAL	WITH proANP	WITHOUT proANP	P*

No. of participants	2498	2203	295	

Age, years	52.7 ± 9.5	52.7 ± 9.4	53.0 ± 10.7	0.566

Sex, male (%)	962(38.51)	842(38.22)	120(40.68)	0.453

Education, high school or above (%)	507(20.30)	468(21.24)	39(13.22)	0.002

Current smoking, n(%)	582(23.30)	500(22.70)	82(27.80)	0.061

Current drinking, n(%)	465(18.62)	417(18.93)	48(16.27)	0.307

Anti-hypertensive medication, n(%)	623(24.94)	554(25.15)	69(23.39)	0.560

Body mass index, kg/m^2^	24.78 ± 3.63	24.80 ± 3.68	24.65 ± 3.26	0.464

Fasting glucose, mmol/L	5.40 ± 1.34	5.42 ± 1.36	5.25 ± 1.17	0.004

Total cholesterol, mmol/L	5.22 ± 1.75	5.21 ± 1.57	5.22 ± 2.75	0.118

Triglycerides, mmol/L	1.46 ± 1.59	1.48 ± 1.63	1.35 ± 1.21	0.057

LDL cholesterol, mmol/L	3.00 ± 0.76	2.99 ± 0.75	3.04 ± 0.86	0.393

HDL cholesterol, mmol/L	1.51 ± 0.44	1.51 ± 0.45	1.49 ± 0.41	0.370

proANP, nmol/L	–	1.20 ± 0.80	–	–


All results are expressed with mean ± SD unless otherwise noted. LDL: low-density lipoprotein; HDL: high-density lipoprotein; ANP: atrial natriuretic peptide. * Comparison between participants with and without proANP at baseline.

### Association between serum proANP and incident CVD

**Figure 2:**
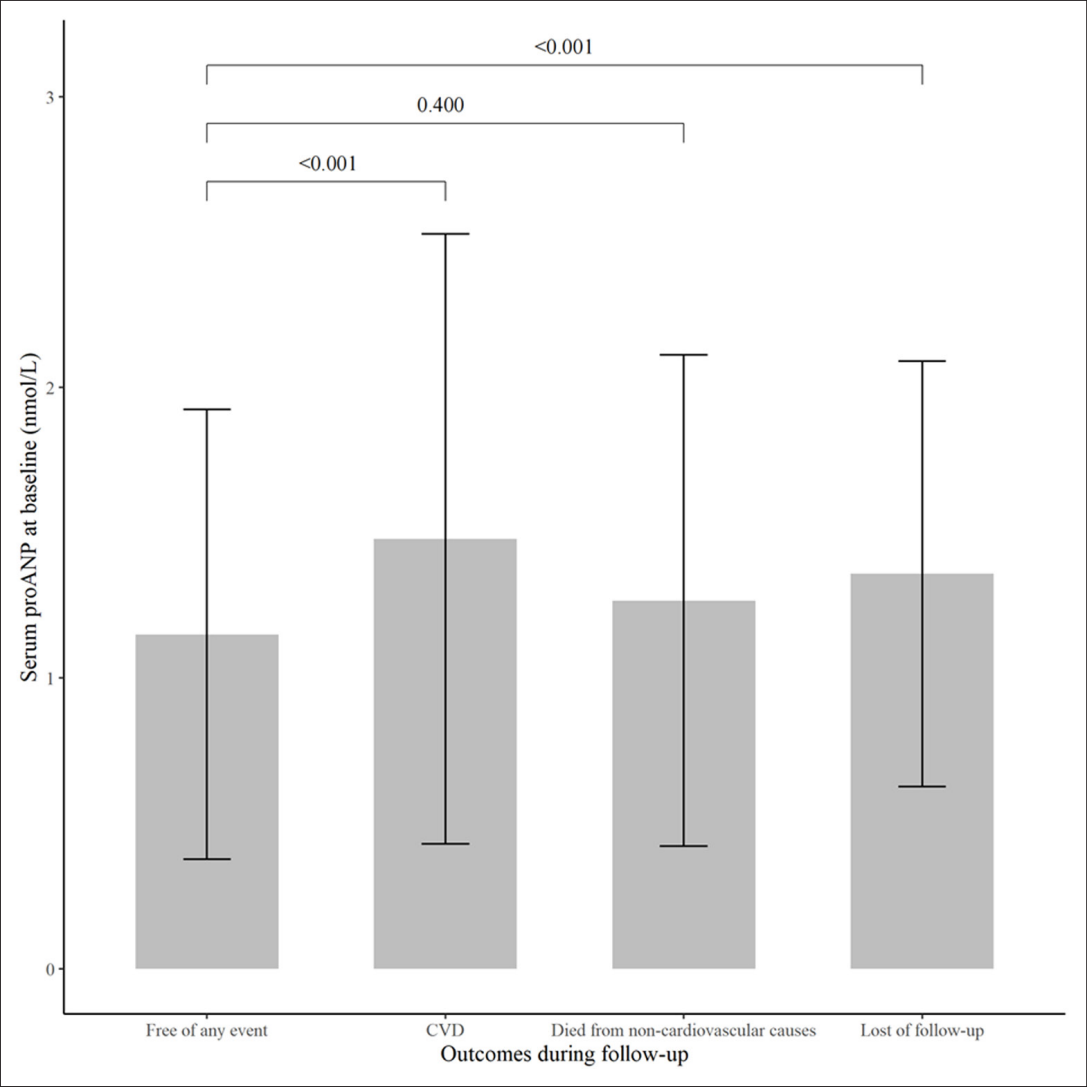
Serum proANP levels for participants with different outcomes during follow-up. Compared to participants who remained free of CVD (mean ± SD: 1.15 ± 0.77 nmol/L), those who died from non-cardiovascular causes (mean ± SD: 1.27 ± 0.84 nmol/L, *P* = 0.400) had a similar level of serum proANP, whereas those who developed CVD (mean ± SD: 1.48 ± 1.05 nmol/L, *P* < 0.001) or were lost during follow-up (mean ± SD: 1.36 ± 0.73 nmol/L, *P* < 0.001) had a significantly increased level of serum proANP at baseline. CVD: cardiovascular disease.

**Figure 3:**
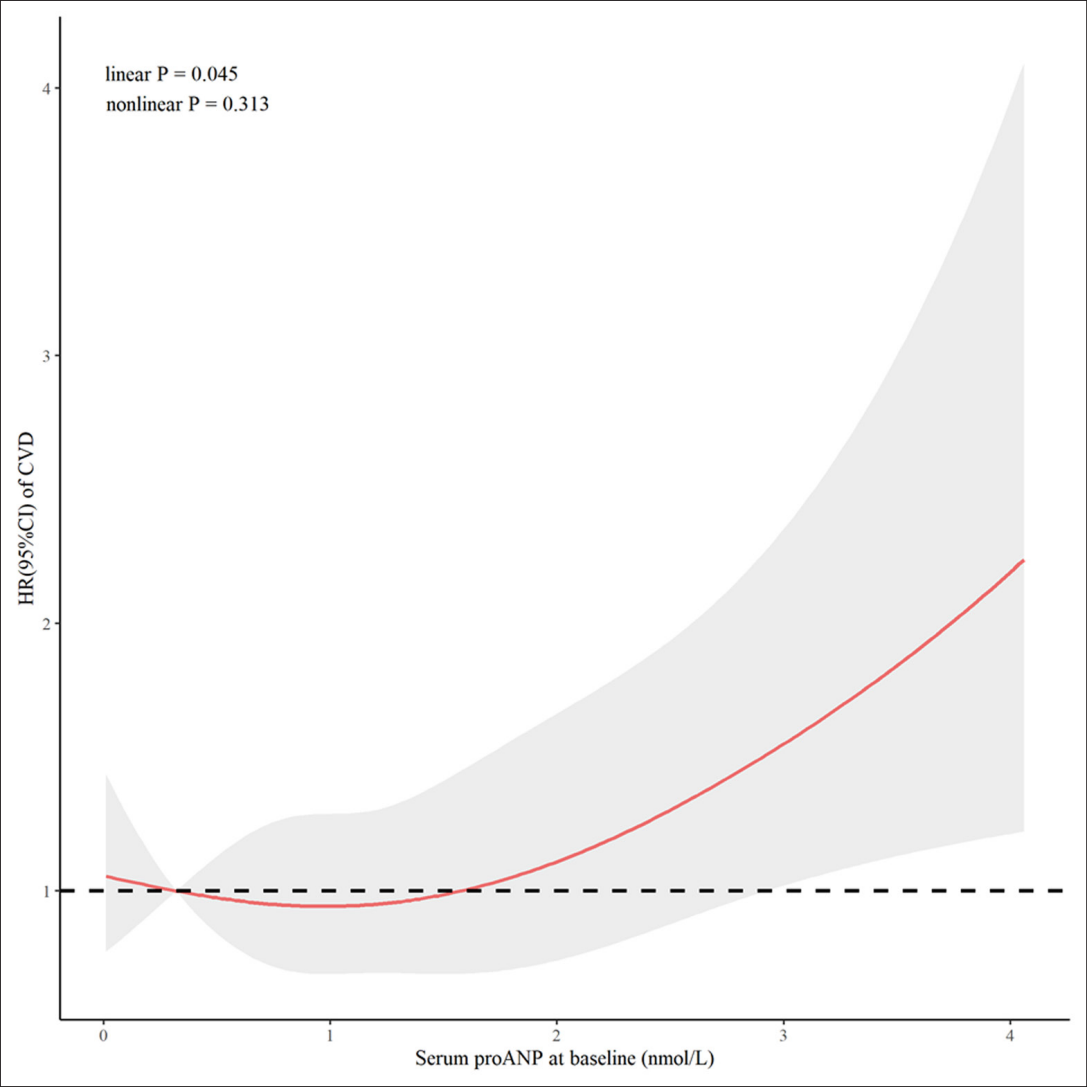
Cubic spline curves visualizing the impact of serum proANP on CVD. Hazard ratios (red line) and their 95% confidence intervals (gray shadow) of CVD associated with baseline serum proANP levels were calculated by constructing a restricted cubic spline regression model with knots placed at the 5^th^, 35^th^, 65^th^, and 95^th^ percentiles of serum proANP levels, after adjusting for age, sex, education level, current smoking, current drinking, systolic blood pressure, body mass index, low-density lipoprotein cholesterol, high-density lipoprotein cholesterol, fasting glucose, and antihypertension medications at baseline. HR: Hazard ratios.

Among 2,203 participants with available data on proANP at baseline, 181 participants developed CVD events, 39 participants died from non-cardiovascular causes, and 197 participants were lost (follow-up rate of 91.06%) during an average of 10 years of follow-up. Their average levels of serum proANP at baseline are shown in ***Figure 2***. Compared to participants free of any outcomes during follow-up, those who developed CVD had a significantly increased level of proANP (mean: 1.48 vs. 1.15 nmol/L, *P* < 0.001). As illustrated in ***Figure 3***, serum proANP at baseline was linearly associated with an increased risk of CVD during follow-up (*P* = 0.045 for linearity test). A 1-nmol/L increment of serum proANP was associated with a 49% (HR = 1.49, 95%CI: 1.26–1.75, *P* < 0.001) and 22% (HR = 1.22, 95%CI: 1.03–1.44, *P* = 0.025) higher risk of CVD during follow-up before and after adjustment for age, sex, education level, cigarette smoking, alcohol consumption, BMI, fasting glucose, LDL-C, HDL-C, SBP, and antihypertensive medications at baseline, respectively (***Table 2***). Compared to participants with a lower level of serum proANP (lower 3 quartiles), those with the highest quartile of serum proANP had an 84% (HR = 1.84, 95%CI: 1.36–2.48, *P* < 0.001) higher risk of CVD (***Table 2***). This association was bottom-line significant after adjusting for conventional risk factors at baseline (HR = 1.34, 95%CI: 0.99–1.81, *P* = 0.059).

**Table 2:** The prospective association between baseline proANP and CVD events during follow-up.


SUBGROUPS	UN-ADJUSTED		ADJUSTED*	
	
	HR (95% CI)	*P*	HR (95% CI)	*P*

Continuous				

proANP (per 1 nmol/L)	1.49 (1.26–1.75)	<0.001	1.22 (1.03–1.44)	0.025

Categorical				

Lower (<1.61 nmol/L)	1.00 (ref)	–	1.00 (ref)	–

Higher (≥1.61 nmol/L)	1.84 (1.36–2.48)	<0.001	1.34 (0.99–1.81)	0.059


* Adjusted for age, sex, education level, cigarette smoking, alcohol consumption, body mass index, fasting glucose, and low- and high-density lipoprotein cholesterol, systolic blood pressure, and antihypertensive medication at baseline. CVD: cardiovascular disease; HR: Hazard ratio; CI: Confidence interval.

### Association between *NPPA* promoter methylation and incident CVD

**Figure 4:**
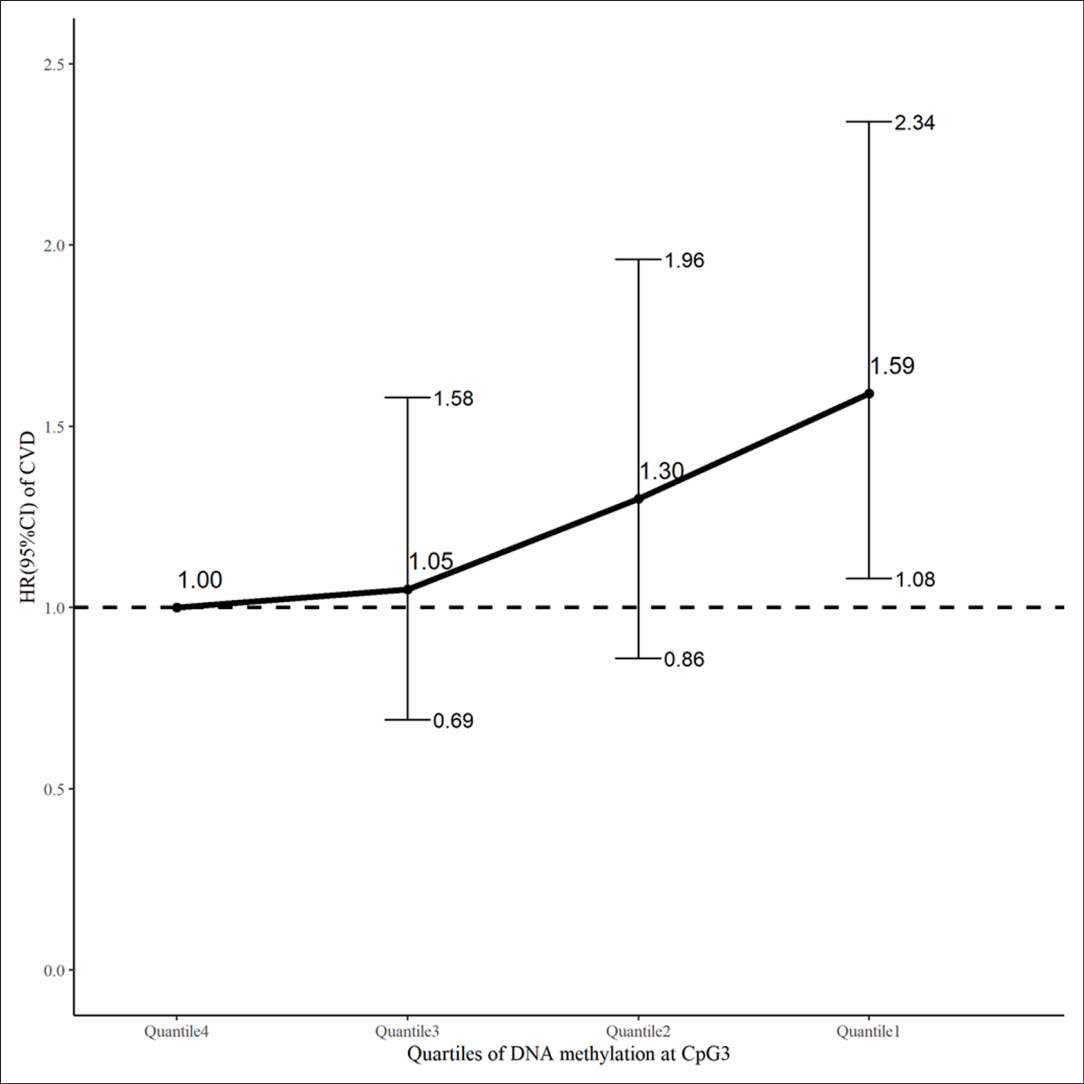
An illustration of the association between quartiles of DNA methylation level at CpG3 (with the highest quartile as a reference) and incident CVD. Hazard ratios (black dot) and their 95% confidence intervals (error bar) of CVD for participants with the 1^st^, 2^nd^, and 3^rd^ quartiles, in comparison to those with the highest quartile of CpG3 methylation, were calculated by a competing-risks survival regression model, adjusting for age, sex, education level, current smoking, current drinking, systolic blood pressure, body mass index, low- and high-density lipoprotein cholesterol, fasting glucose, and antihypertension medications at baseline.

Among 2,498 participants with available data on *NPPA* methylation, 210 participants developed CVD events, 50 participants died from non-cardiovascular causes, and 214 participants were lost (follow-up rate of 91.43%) during follow-up. Of the 9 CpG sites assayed, a higher level of DNA methylation at CpG3 (located at Chr1:11908299, –459 bp relative to TSS) was significantly associated with a lower risk of CVD (HR = 0.50, 95%CI: 0.30–0.82, *P* = 0.006 for log2-transformed methylation), after adjusting for age, sex, education level, cigarette smoking, alcohol consumption, BMI, fasting glucose, LDL-C, HDL-C, SBP and antihypertensive medications at baseline (***Table 3***). After further correction for multiple testing, this association was still survived (q = 0.051). Regression using quartiles of DNA methylation level at this CpG site as the independent variable found that participants with the lowest quartiles had a 59% (HR = 1.59, 95%CI: 1.08–2.34, *P* = 0.019) increased risk of CVD, compared to those with the highest quartile (***Figure 4***).

**Table 3:** The prospective association between baseline *NPPA* promoter methylation and incident CVD.


CpG loci	GENOMIC POSITION, GRCh37	RELATIVE TO TSS, bp	AVERAGE METHYLATION %	INCIDENT CVD		

				HR (95%CI)*	*P*	q

Single CpG association						

CpG1	Chr1:11908353	–513	28.55 ± 5.22	0.67 (0.40–1.12)	0.129	0.340

CpG2	Chr1:11908348	–508	93.17 ± 2.52	0.89 (0.02–32.47)	0.950	0.950

CpG3	Chr1:11908299	–459	22.84 ± 3.84	0.50 (0.30–0.82)	0.006	0.050

CpG4	Chr1:11908200	–360	68.28 ± 6.49	0.60 (0.23–1.57)	0.294	0.529

CpG5	Chr1:11908182	–342	81.68 ± 4.89	0.49 (0.10–2.36)	0.378	0.566

CpG6	Chr1:11908178	–338	40.06 ± 6.12	0.66 (0.37–1.17)	0.151	0.340

CpG7	Chr1:11908168	–328	50.29 ± 6.44	0.53 (0.26–1.07)	0.077	0.340

CpG8	Chr1:11908165	–325	30.68 ± 6.41	0.89 (0.59–1.32)	0.552	0.710

CpG9	Chr1:11908142	–302	36.53 ± 7.74	0.91 (0.62–1.34)	0.633	0.712

Gene-based association						

wTPM					0.008	


Risks of incident CVD associated with every twofold increase in DNA methylation levels during follow-up, after adjusting for age, sex, education level, cigarette smoking, alcohol consumption, body mass index, fasting glucose, and low- and high-density lipoprotein cholesterol, systolic blood pressure, antihypertension medication. CVD: cardiovascular disease; GRCh37: Genome Reference Consortium Human Build 37; TSS: Transcription start site; HR: Hazard ratio; CI: Confidence interval; wTPM: Weighted truncated product method.

Although we failed to find a statistically significant association between DNA methylation at other CpG sites and incident CVD, the wTPM revealed that DNA methylation at these 9 CpG sites as a whole was significantly associated with incident CVD (all *P* = 0.008, ***Table 3***). GTEx database showed that the *NPPA* gene was not only expressed in white blood cells but also expressed in artery and heart. iMethyl database showed that DNA methylation at all of the 9 CpG sites assayed could occur in white blood cells but none of them were expression quantitative trait methylation (eQTMs).

### Association between *NPPA* promoter methylation and serum proANP

**Figure 5:**
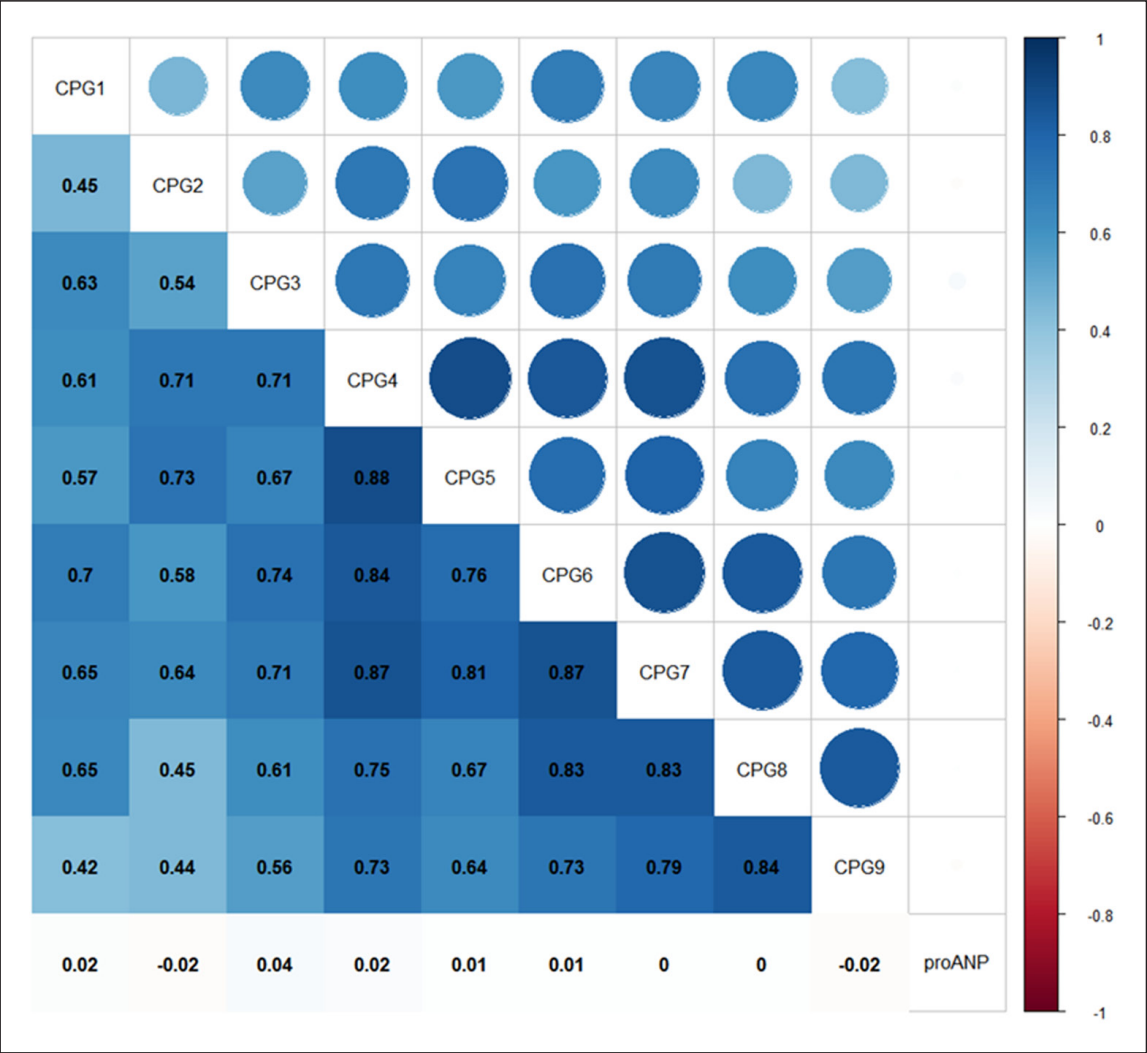
The spearman correlation matrix among DNA methylation levels at all CpG loci assayed in *NPPA* promoter and proANP. The spearman correlation coefficient is shown in the lower left part of the picture. The color depth of the square represents the strength of the correlation. The size and color of the circle in the upper left part of the picture also represent the strength of correlation. Large circle area and color represent strong correlation.

***Figure 5*** illustrates the correlation matrix among each CpG methylation and serum proANP. As expected, DNA methylation levels at neighboring CpG sites were highly correlated. Nevertheless, we did not find any CpG sites whose methylation level significantly correlated to serum proANP.

## Discussion

In a prospective cohort study including 2498 middle-aged and elderly Chinese adults, our study for the first time examined the prospective associations of proANP and *NPPA* promoter methylation levels with incident CVD in the Chinese Han population. The results showed that a higher level of serum proANP at baseline, but a lower level of DNA methylation of *NPPA* promoter, significantly predicted a higher risk of incident CVD during an average of 10 years of follow-up. Although we failed to find a negative correlation between *NPPA* promoter methylation assayed in our study and serum proANP, our results may indicate that DNA methylation of the coding gene may be a molecular mechanism underneath the effect of ANP on CVD development. In light of these findings, *NPPA* promoter methylation may possess the potential to serve as a predictor, or even probably a therapeutic target, for CVD.

In line with our study, the observed association between serum proANP elevation and increased risk of CVD has also been found by prior studies. For example, a prospective study including 5,130 middle-aged and elderly adults found that higher plasma levels of proANP were associated with the incidence of atrial fibrillation and stroke during a median follow-up of 5.6 years [9]. Clinical trials also demonstrated the effect of ANP on the development of CVD. For instance, a randomized clinical trial including 277 patients with acute myocardial infarction in the treatment arm and 292 patients in the placebo group found that intravenous ANP (0.025 microg/kg per min for 3 days) could reduce infarct size [10]. However, synthetic human ANP has some adverse effects such as severe hypotension [13,14,15] and in-hospital death [16,17,18]. A better understanding of the underlying molecular mechanisms would help clinical translation of ANP. In addition to ANP, its coding gene (*NPPA*) has also been suggested by previous studies. For example, a polymorphism in the *NPPA* gene (rs5065) has been associated with CHD [23,38], atherosclerosis [39], and ischemic stroke [2]. As an interface between the fixed genome and dynamic environment, epigenetic modifications such as DNA methylation play a crucial role in the regulation of transcriptional activity and gene expression [25], and thereby participating in the development of CVD. Therefore, we further examined whether DNA methylation of the coding gene of ANP (*NPPA*) at baseline could predict the risk of CVD incidence in the Gusu cohort.

Indeed, this epigenetic regulation of ANP expression has been observed by integrative analyses on DNA methylomes and transcriptomes data in humans [40]. Some CVD-related methylation markers have been found in animals and humans. For example, in atherosclerosis-prone apolipoprotein E (ApoE)-null mice, DNA methylation changes occurred in both peripheral blood leukocytes and the aorta prior to the formation of vascular lesions [41]. In the Normative Aging Study conducted in the Boston area, participants with ischemic heart disease and stroke had lower blood Long Interspersed Nucleotide Element-1 (*LINE-1*) methylation, while in longitudinal analyses, lower *LINE-1* methylation was associated with a higher risk of ischemic heart disease and stroke [42]. Numerous EWAS studies have been conducted and identified many epigenetic markers of CVD [27,28], but no study, to the best of our knowledge, has reported the role of DNA methylation at the *NPPA* gene in CVD. Leveraging an unselected population in the Gusu cohort, we are the first to examine the prospective associations of the *NPPA* promoter methylation with CVD and provide initial evidence for the potential role of the *NPPA* promoter methylation in the pathogenesis of CVD.

In this study, we also found that the contribution of an individual CpG methylation to CVD was in general small (mostly <5%), and statistically most CpG sites could not withstand multiple testing correction. Such a small effect size may not be detected by conventional statistical methods, but their combined effects may be large enough to be useful for risk prediction. Therefore, we tested the joint association of multiple CpG methylation with CVD and found that the joint contribution of these CpG sites appeared to be much larger. Our results may unravel a molecular mechanism that the *NPPA* promoter methylation may participate in the pathology of CVD, and suggest that simultaneously testing the joint effects of multiple CpG sites is a powerful approach in epigenetic analysis for complicated diseases, e.g., CVD.

To the best of our knowledge, our study is the first to examine the prospective association of *NPPA* promoter methylation with CVD in Chinese adults. The strengths of this study include a prospective longitudinal study design of the associations of *NPPA* promoter methylation with CVD, comprehensive adjustments of many conventional risk factors, and application of a gene-based analytical approach to testing the combined effect of multiple CpG methylation at *NPPA* promoter on CVD. However, our study also has several limitations. First, as in all observational studies, unobserved confounders may exist in our study. Second, we only included middle-aged and elderly Chinese adults in our study. The generalizability of our findings to other age groups or populations with different ethnic backgrounds is uncertain. Third, given that DNA methylation is tissue- and cell-type specific, it is unclear whether or to what extent the results derived from peripheral blood could reflect methylation changes in the target organs of CVD. However, accumulating evidence indicated that epimutations may not be limited to the affected tissue but could also be detected in peripheral blood [43,44,45]. Fourth, this epigenetic regulation of ANP expression has been observed by integrative analyses on DNA methylomes and transcriptomes data in humans [40]. However, we did not find an association between *NPPA* promoter methylation and serum proANP in our study. Nevertheless, our results may suggest that the assayed CpG sites, CpG 3 in particular, could be a potential biomarker for the risk of CVD.

## Clinical perspectives

ANP has been associated with cardiovascular disease, but the clinical translation is limited. A better understanding of the molecular mechanisms of the cardiovascular effect of ANP will help its drug development and improvement. As an interface between the fixed genome and dynamic environment, epigenetic modifications such as DNA methylation play a crucial role in the regulation of transcriptional activity and gene expression, and thereby participating in the development of CVD. Our study demonstrated that hypomethylation at *NPPA* promoter at baseline predicted an increased risk of future CVD in Chinese adults. These findings indicate that *NPPA* promoter methylation could serve as a predictor for the identification of individuals at high risk for CVD during primary prevention, but more evidence is needed to establish the causality between *NPPA* promoter methylation and CVD.
